# Clinicopathological and molecular genetic alterations in monomorphic–epitheliotropic intestinal T-cell lymphoma of the small intestine

**DOI:** 10.1186/s40001-024-01797-5

**Published:** 2024-03-23

**Authors:** Bing Zhou, Min Guo, Xiaohua Li, Ting Duan, Lizi Peng, Hua Hao

**Affiliations:** 1 Department of Pathology, Second Affiliated Hospital of Jiujiang University, Jiujiang, 332005 Jiangxi People’s Republic of China; 2 Department of Pathology, Seventh People’s Hospital of Shanghai University of TCM, Shanghai, 200137 People’s Republic of China; 3 Department of General Surgery, Second Affiliated Hospital of Jiujiang University, Jiujiang, 332005 Jiangxi People’s Republic of China; 4 Department of Pathology, Zhejiang Provincial People’s Hospital, Hangzhou, 314408 Zhejiang People’s Republic of China; 5 Department of Pathology, Jiujiang First People’s Hospital, Jiujiang, 332000 Jiangxi People’s Republic of China; 6 Department of Pathology, Yangpu Hospital, School of Medicine, Tongji University, 450 Tengyue Road, Shanghai, 200090 People’s Republic of China

**Keywords:** MEITL, TCR, NGS, JAK3, STAT5B, SETD

## Abstract

**Background:**

Small intestinal monomorphic–epitheliotropic intestinal T-cell lymphoma (MEITL) is a rare aggressive T-cell lymphoma originating in the gastrointestinal tract. This study aimed to investigate the clinicopathological features, immunophenotypes, and molecular genetic changes of MEITL.

**Methods:**

The clinicopathological data for three patients with surgically resected MEITL of the small intestine were collected. Next, immunohistochemical labeling, Epstein–Barr virus (EBV) in situ hybridization, assessment of clonal rearrangement of T-cell receptor (TCR) genes, and next-generation sequencing (NGS) were performed.

**Results:**

Of the three patients, two were male and one was female, with ages of 61, 67, and 73 years, respectively. Clinical manifestations were predominantly abdominal pain and distension. Histopathology revealed infiltrative growth of small-to-medium-sized lymphocytes with a consistent morphology between the intestinal walls, accompanied by an obvious pro-epithelial phenomenon. The expression of CD3, CD8, CD43, CD56, TIA-1, CD103, H3K36me3, and Bcl-2 was detected, and the Ki-67 proliferation index ranged from 50% to 80%. All three patients tested negative for EBER. However, monoclonal rearrangement of the TCR gene was detected in them. NGS testing showed a *JAK3* mutation in all three cases. Further, *STAT5B*, *SETD2*, and *TP53* mutations were each observed in two cases, and a *BCOR* mutation was found in one case. All patients were treated with chemotherapy after surgery. Two patients died 7 and 15 month post-operation, and one patient survived for 5 months of follow-up.

**Conclusions:**

Our findings demonstrate that mutations in *JAK3* and *STAT5B* of the JAK/STAT pathway and inactivation of the oncogene *SETD2* markedly contribute to the lymphomagenesis of MEITL.

## Background

Monomorphic–epitheliotropic intestinal T-cell lymphoma (MEITL) is a rare extranodal T-cell lymphoma originating from T-lymphocytes within the epithelium of the gastrointestinal tract and is most reported in Asia. It accounts for less than 1% of all non-Hodgkin’s lymphoma cases. Although it can also occur in the stomach and colorectum, it is most common in the small intestines (63%). Currently, it is recognized as a separate entity based on its distinct clinicopathological and epidemiological features, which are unrelated to those of celiac disease (CD). The World Health Organization (WHO) 2016 edition of the classification of tumors of the lymphohematopoietic system classified type II enteropathy-associated T-cell lymphoma (EATL) as a separate subtype formally named MEITL. Furthermore, studies have confirmed that this lymphoma has distinctive origins, pathological features, and molecular genetics [1]. In this study, we analyzed the clinicopathological features and immunophenotypic and molecular genetic changes in three patients with MEITL of the small intestines and reviewed the relevant literature to improve our understanding of MEITL.

## Methods

### Data

Three patients were diagnosed with MEITL of the small intestines at Jiujiang First People’s Hospital of Jiangxi Province and Zhejiang Provincial People’s Hospital. MEITL specimens were surgically resected and collected from June 2019 to February 2023. Then, the clinical, imaging, and laboratory examination data as well as pathological data of the patients were reviewed. Moreover, follow-up information was obtained telephonically. All patients met the MEITL diagnostic criteria outlined in the 2016 and 2022 editions of the WHO classification criteria for lymphohematopoietic system tumors.

### Morphological observation and immunohistochemistry

The histological specimens from the three patients were fixed, sampled, dehydrated, embedded, sectioned, and stained with hematoxylin and eosin (HE). Then, morphological observations were performed under a light microscope. Immunohistochemical staining against the antibodies: anti-CD3, anti-CD4, anti-CD5, anti-CD8, anti-CD20, anti-CD79α, anti-CD10, anti-CD30, anti-CD43, anti-CD56, anti-TIA-1, anti-Granzyme B, anti-CD103, anti-H3K36me3, anti-Bcl-2, anti-CK, and anti-Ki-67 from Beijing Zhongsui Jinqiao (Beijing, China) was performed. Two senior pathologists reviewed all the slides.

### In situ hybridization

Three paraffin tissue samples were sectioned into 3.0 μm pieces, dewaxed, hydrated, digested with pepsin, dehydrated, dried, and incubated with digoxigenin-labeled Epstein–Barr virus encoding microRNA (EBER) probe for 3–4 h. Drops of HRP-labeled anti-digoxigenin antibody were added, and the samples were incubated for 30 min. The samples were washed with PBS, and the color was developed using DAB. They were subsequently rinsed with running water, re-stained with hematoxylin, differentiated with ethanol hydrochloride, re-blued with ammonia, and blocked after dehydration. The hybridization kit was purchased from Beijing Zhongshan Jinqiao Biotechnology Co (Beijing, China). Positive staining was localized in the nucleus, which was brownish-yellow.

### T-cell receptor (TCR) gene (TCRB + TCRG) rearrangement detection

DNA was extracted from the tumor tissues of three patients using a nucleic acid extraction kit and purified using the QIAamp DSP DNA FFPE tissue kit (Giagen, Hilden, Germany). A PCR instrument (ABI, Waltham, MA, United States) was then utilized for DNA amplification using the Shanghai Yuan Qi amplification kit (Shanghai, China). The amplified samples were tested for gene rearrangement, and the checkpoints included Vβ + Jβ, Dβ + Jβ fragments of TCRβ, Vγ1f, Vγ10–Jγ, Vγ9, Vγ11–Jγ fragments of TCRγ.

### Next-generation sequencing (NGS)

DNA was extracted from tumor tissues of the three patients, and DNA sequencing libraries were constructed using a library preparation kit (Illumina, San Diego, CA, United States) with a 66-gene panel probe that is closely related to lymphoma as a gene capture method. Sequencing was performed using the Illumina Hieseq 4000 sequencing platform, with sequencing lengths of up to 150 bp and a sequencing depth of 500 × . The obtained data were bioinformatically filtered and analyzed.

## Results

### General information

**Fig. 1:**
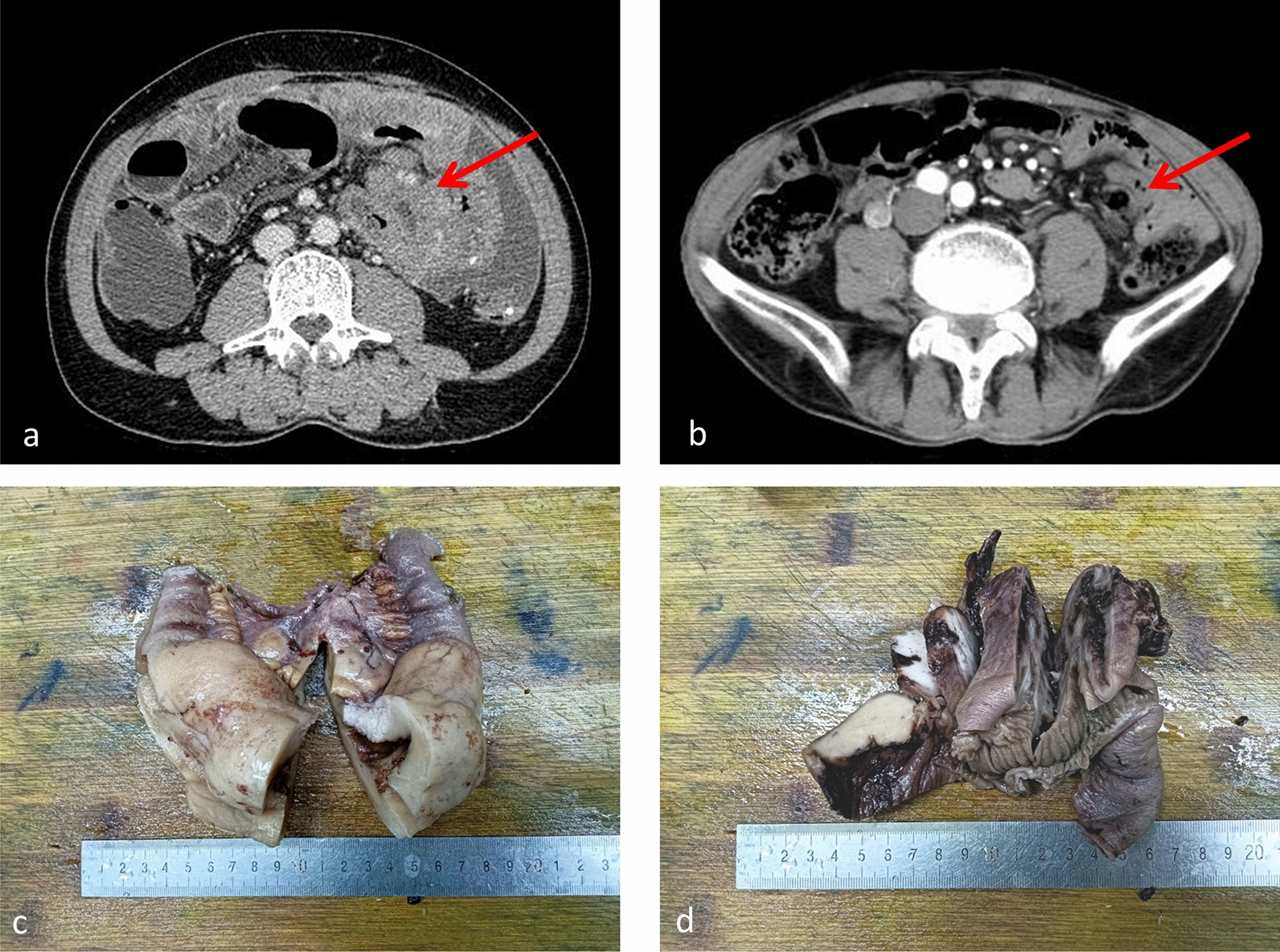
**a** CT showing small bowel occupying lesion; **b** CT showing small bowel occupying with adhesions; **c** large body showing small bowel mass; **d** large body showing small bowel mass

The male-to-female ratio among the three patients with small bowel MEITL was 2:1, and the average age was 67 years (61–73 years). All patients clinically presented with abdominal pain and distension, with two patients reporting intestinal obstruction and bloody stools and one with intestinal perforation. None of the patients had CD. Abdominal CT showed small bowel occupancy in two patients (Fig. 1a) and perforation in one patient, with intestinal adhesions (Fig. 1b). The lesion site was the jejunum in two cases and the ileum in one case. Laboratory tests revealed elevated levels of C-reactive protein, lactate dehydrogenase, and Ca125. Here, the lymphoma was treated by surgical resection supplemented with postoperative chemotherapy. Two patients died of disease progression 7 and 15 months after surgery, whereas one patient remained tumor-free for 5 months (Table 1).

**Table 1:** Clinical and pathological data of 3 cases of small bowel MEITL

Case	Sex/age (years)	symptom	Laboratory tests	Tumor site	Size of swelling (cm^3^)	Initial diagnosis	Interoperative view	Curing	Prognosis (survival time)
1	Female/61	Abdominal distension with obstruction	CRP: 35.9 mg/L LDH: 621 U/L CA125: 401.5U/mL	ileum (anatomy)	13 × 8 × 6	small bowel syndrome	Thickening of the intestinal wall and narrowing of the intestinal canal	Surgery + CHOP	At 17 months of follow-up, the patient died
2	Male/67	Abdominal pain, bloody stool with perforation	CRP: 63.24 UI/L LDH: 524.1U/L CAl25: 186.3 U/mL	empty stomach	3 × 2 × 2	perforated intestine	Ulcerative mass with perforation/bleeding in the intestinal wall	Surgery + CHOP	At 8 months of follow-up, the patient died
3	Female /73	Epigastric distension with obstruction	CRP: 22.7 mg/L LDH: 247 U/L CAl25: 106.7 U/mL	empty stomach	5 × 3 × 2	Small bowel occupation with obstruction	Hard mass in the intestinal wall	Surgery + CPCT + Chinese medicine	Alive (5 months)

LDH: (106–300 U/L), CRP: (0–5.0 mg/L), CA125: (0–35 U/mL). CHOP: cyclophosphamide + vincristine + epirubicin + prednisone. *CPCT* cedarbenzamide, prednisone, cyclophosphamide, thalidomide

### Pathological features

**Fig. 2:**
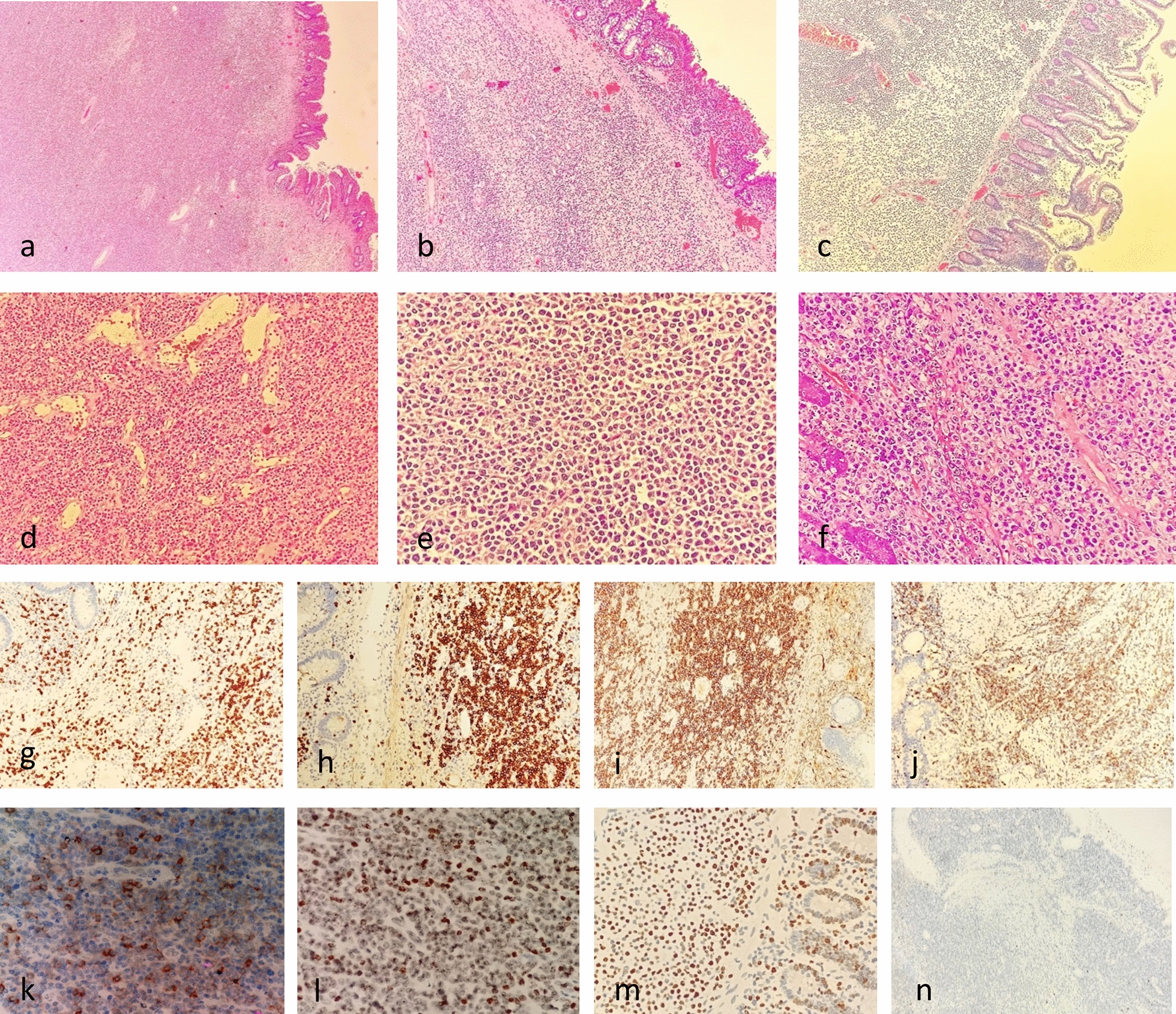
**a** Infiltrative growth of submucosal tumour cells; **b** ‘‘pro-epithelial phenomenon’’ of tumour cells; **c** marked increase in lymphocytes within the largely normal intestinal mucosa; **d** hyperplasia of endothelial veins in tumour tissues; **e** tumour cells are more consistent in morphology, small-medium in size; **f** tumour cells are medium in size with heterogeneity, nuclear fission and fragmentation are seen; **g** tumour cell CD3 cytoplasmic and cytosolic positivity; **h** tumour cell CD8 cytosolic and cytosolic positivity; **i** tumour cell CD56 cytosolic positivity; **j** tumour cell TIA-1 cytoplasmic positivity; **k** tumour cell CD103 cytosolic positivity; **l** Tumour cell H3K36me3 cytosolic positivity; **m** tumour cell Ki-67 proliferation index ranged from 50% to 80%; **n** tumour cell EBER negativity

The tissues sent for examination were all small intestinal bowel resection specimens of 9–27 cm in length and 2.5–4 cm in diameter. The largest diameter of the mass was 3–13 cm, with greyish-white and greyish-brown solid and medium texture on the sectioned surface. Furthermore, there were unclear boundaries, and no necrosis was observed (Fig. 1c). These pathological features were accompanied by intestinal perforation in one patient (Fig. 1d). Light microscopy revealed diffuse and destructive growth of lymphoid tumor cells in the submucosa of the small intestines (Fig. 2a). The tumor cells had a clear “pro-epithelial phenomenon” that was characterized by atrophy and deformation of the intestinal mucosa, with destruction of crypts (Fig. 2b). In some areas, the intestinal mucosa was intact, and the villi were mildly atrophied, with a marked increase in intraepithelial lymphocytes (Fig. 2c). Moreover, the morphology of the tumor cells was relatively uniform, and endothelial venous hyperplasia was observed in the tumor area without considerable necrosis (Fig. 2d). The tumor cells were small- to medium-sized, round, and ovoid, with a pale cytoplasm, centered nuclei, fine chromatin, and invisible nucleoli (Fig. 2e). Some of the tumor cells in Case 2 were medium-sized, with increased cellular heterogeneity, vacuolated nuclei, visible nuclear division, and nuclear fragmentation (Fig. 2f). Immunohistochemical markers CD3 (Fig. 2g), CD8 (Fig. 2h), CD43, CD56 (Fig. 2i), TIA-1 (Fig. 2j), CD103 (Fig. 2k), H3K36me3 (Fig. 2l), and Bcl-2 were positively expressed, whereas granzyme B (2/3) and CD30 (1/3) were partially expressed. Contrastingly, CD4, CD5, CD20, CD79α, CD10, and CKpan were negatively expressed. The Ki-67 proliferation index ranged from 50% to 80% (Fig. 2m).

### Molecular genetic characteristics

In situ hybridization of small RNA encoded by EBV was negative in all three cases (Fig. 2n). However, TCR gene rearrangement was positive in all three patients with MEITL, with monoclonal rearrangement of TCRG (TCR-γ) in two cases and that of TCRB (TCR-β) in one case. Furthermore, mutation of *JAK3* was detected through NGS in all three specimens, with *STAT5B*, *SETD2*, and *TP53* mutations in two cases each. A *JAK3* mutation was detected in all three cases. This was accompanied by mutations of each of *STAT5B*, *SETD2*, and *TP53* in two patients, whereas BCOR gene mutation was detected in only one case. The mutation types were mainly point and code-shift mutations (Table 2).

**Table 2:** Molecular genetic changes of MEITL in small intestine in 3 cases

Case	EBER	TCR gene rearrangement	NGS			
			Gene	Mutant Type	Mutant amino acid changes	Mutant abundance (%)
1	–	TCR⁃γ	JAK3	Missense	p.A573V	17.3
			SETD2	Frameshift indel	p.D2004I	13.2
			TP53	Missense	p.R248Q	24.7
			BCRO	Frameshift indel	p.L1262	6.5
2	–	TCR⁃β	JAK3	Missense	p.V674A	22.1
			SETD2	Missense	p.S1769T	9.4
			STAT5B	Missense	p.Y655F	6.9
			TP53	Frameshift indel	p.I232S	16.3
3	–	TCR⁃γ	JAK3	Missense	p.A573V	36.7
			STAT5B	Missense	p.N642H	11.8

## Discussion

Less than 50 cases of primary small-bowel MEITL have been reported worldwide since the 2016 official WHO classification of tumors of hematopoietic and lymphoid tissues. These tumors are more prevalent in Asian populations, with a median onset age of approximately 60 years and a male-to-female incidence ratio of approximately 2:1. In this study, the age of the three patients was > 60 years, which is in line with the literature reports. Furthermore, two of the patients were female, which is slightly different from previous studies [2]. The disease onset can involve the entire small intestines. However, the ileum is a more common site of onset. In this study, the site of onset in two cases was consistent with the existing literature [3, 4]. Unlike type I EATL, CD is extremely rare. Additionally, early stage MEITL often manifests as abdominal pain, abdominal distension, and other intestinal nonspecific symptoms that are easily misdiagnosed as inflammatory bowel disease [5]. Obstruction or perforation can easily occur in the late stage. The three patients enrolled herein were all in the late stage, suggesting that the onset of the disease is insidious and that the clinical course is long. The mild edema, punctate erythema, and superficial erosion with ulceration and peripheral destruction of villous structures observed under balloon endoscopy can improve the detection rate of MEITL [6]. However, laboratory tests for patients with MEITL remain controversial. Most scholars believe that CRP, LDH, and CA125 elevation detected by serology is suggestive of the disease. However, other reports consider these features less meaningful. The laboratory findings in this study were consistent with the former observation, potentially due to the high load of lymphoma in the progressive stage and the damage to the whole layer of the intestinal canal, which predicts a worse prognosis [7, 8].

Most MEITL patients present with acute symptoms, mainly related to intestinal perforation and/or obstruction by an infiltrative tumor mass [9]. Early stage gross morphology is not specific, but the late stage involves the whole layer of the bowel wall, visible mucosal ulceration, thickening and narrowing of the intestinal lumen, or perforation. The three patients in this study were in the advanced stage of the disease, presenting diffuse proliferative mass of the bowel wall, visible remnants of the intestinal mucosa, greyish-white solid cut section with a fine texture, and rare necrosis. The histological pattern was similar to that of MEITL in other parts of the digestive tract, showing diffuse growth of small-to-medium-sized tumor cells with consistent intramucosal and submucosal morphology, a relatively homogeneous background, rare necrosis, small rounded cells, pale-stained cytoplasm, and atrophy of the small intestinal villi with epithelial disruption and crypts. In contrast, there was no obvious “epithelium-philicity” in the adjacent normal mucosa. In this study, two cases had medium-sized tumors, with greater heterogeneity and more nuclear divisions. However, they did not possess large cellular MEITL, as reported in the literature. Pleomorphic and mesenchymal features of the nuclei were also observed [10]. Immunohistochemistry of MEITL tumor cells usually detects CD3, CD7, CD8, CD43, and CD56 expression. Granzyme B, TIA-1, and perforin have been reported to exhibit variable expression in various reports, but are considered to have an activating cytotoxic phenotype. Furthermore, their positive expression positively correlates with the depth of tumor infiltration [11]. This is consistent with the results of the present collection of cases, where the tumors were in the advanced stages. Moreover, positive results were occasionally obtained for CD4 and CD5 but their clinical significance remains unclear. Some studies have reported that the paradoxical expression rate of CD20 in MEITL tumor cells is up to 20%, which was mostly observed in cases with high tumor cell heterogeneity and invasiveness. This also predicted a worse prognosis. However, these findings could be further validated using large samples at a later stage [1]. The proliferation index of Ki-67 was higher, generally > 50%, which also indicates the aggressive biological behavior of this tumor [12]. Immunohistochemical markers detected in the patients enrolled herein were consistent with those in the literature, with no paradoxical expression of CD4, CD5, and CD20, which may be related to the more homogeneous cellular morphology and the small number of cases. The expression of CD103 (the α E subunit of the heterodimer integrin αEβ7), which is characteristic of intraepithelial lymphocytes of the small intestine and documented in T-cell lymphomas, particularly EATL [13], was positive in most cases. Consistently, Veloza et al. [14] reported that among 71 European MEITL patients, 80% exhibited CD103 expression in tumor cells. The loss of H3K36 trimethylation, a hallmark of MEITL, confers high sensitivity to WEE1 kinase inhibitors [15]. Therefore, we examined H3K36me3.

MEITL is not associated with EBV infection. Therefore, tumor cells are negative for EBER expression, and occasional scattered B-cell positivity next to tumor cells should be carefully identified. MEITL of the small intestine needs to be differentiated from Crohn’s disease in the early stages in terms of clinical and endoscopic manifestations, and from EATL, peripheral T-cell lymphoma of the gastrointestinal tract, intestinal inert T-lymphoproliferative disorders, NK/T-cell lymphoma, and B-cell lymphoma in the late stages. These are lymphoid disorders occurring in the small intestine and are associated with the morphology of the tumor cells at the microscopic level, specific immunohistochemical markers, and negativity for EBERs. Features, such as EBER negativity, can help us make a final diagnosis.

TCR gene rearrangement is of great significance in the diagnosis of T-cell lymphoma. Approximately 80% of cases express TCRβ and TCRγ. MEITL derived from T cells expresses TCRγ rearrangements more often. However, absence of TCRγ and TCR expression has also been reported [10, 16]. The JAK–STAT pathway plays a role in the oncogenic mechanisms of T-cell lymphomas, NK-cell lymphomas, and leukemia. JAK and STAT molecules contribute to cytoplasmic-to-cytosolic signaling in the cell by binding to receptors and participating in cytokine activation, inactivation, and regulation of T-cell apoptosis [16–18]. Here, mutation sites were detected in the JAK–STAT axis in all three cases, among which *JAK3* and *STAT5B* were mutated in three and two cases, respectively. All the observed mutations were point mutations. *JAK3* is mainly expressed in immune cells and is involved in their development and maintenance of their activity. Furthermore, it is particularly important for signal transducer and activator of transcription (STAT), and *JAK3* mutations often lead to chromosomal combined immunodeficiency disease [19]. *STAT5B* is expressed in several human organs, mediates various cellular ligand-triggered signal transduction pathways, and regulates cell proliferation/apoptosis and DNA damage by modulating the JAK3–STAT5B signaling pathway, which plays an important role in the development of various tumors [20]. Activation of the JAK/STAT pathway is a ubiquitous feature of MEITL. *JAK3* and *STAT5B*, the most frequently mutated genes in this pathway, may be important factors contributing to the development of MEITL and have potential value and significance in the diagnosis and targeted therapy of MEITL [17, 21, 22]. Two patients developed mutations in *TP53* in the MAPK signaling pathway: one with a point mutation and another with code-shift mutation, which can regulate p53 protein. This process can contribute to tumor development. Moreover, *TP53* mutations reportedly indicate a worse prognosis [23]. Two patients had missense and code-shift mutations in *SETD2*. *SETD2* is a histone lysyltransferase that interacts with RNA polymerase II to regulate histone trimethylation and is considered an oncogene in various malignancies. In the presence of *SETD2* mutations, patients with TP53 mutations develop inactive tumors. Therefore, *SETD2* mutations are important for the development of MEITL [24]. Here, there was a case harboring *BCOR* shift mutation. *BCOR*, a homologous oncogene of the transcriptional repressor Bcl-6, is widely expressed in various tissues of the human body. When mutated, it can promote the proliferation of T cells, leading to the occurrence of lymphoma. This gene is considered a reliable genetic marker for T-cell lymphoma [25]. There is no standard and effective treatment option for small-bowel MEITL to date, and surgical resection and chemotherapy are the mainstay clinical treatment. Some scholars have reported that anthracycline-based CHOP treatment regimens combined with the histone deacetylase inhibitors cetamine and pembrolactam can effectively alleviate and control the disease and prolong survival time [3, 26]. Patients with MEITL who underwent autologous stem cell transplantation showed a substantial improvement in the survival rate, along with a considerable reduction in toxic side effects (28). However, small intestinal MEITL is highly aggressive and has a poor prognosis, often recurring within a short period or with abdominal involvement. This cancer can metastasize to the lungs and brain, and the survival time is usually less than 3 years (29, 30). This study reports three advanced cases of small intestinal MEITL. After resection of diseased intestinal segments, two patients were treated with the CHOP regimen and died of disease progression 7 and 15 months after surgery. One patient was treated with four courses of the CPCT regimen after surgery and supplemented with oral antitumor Chinese medicine (the specific details of which are unknown) intermittently during the period. The follow-up time was 5 months, and temporary tumor-free survival was achieved. However, due to the relatively short follow-up time, the specific therapeutic efficacy could not be determined. Therefore, a large sample size is required to study the specific efficacy of the applied therapies. Currently, various drugs that inhibit the activity of the JAK/STAT3 pathway or SETD2-targeted drugs are in the research and development stage or clinical trials, and some have been proven to have antitumor activity against various subtypes of peripheral T-cell lymphomas (31, 32).

Our study had a limitation. We only collected three cases of MEITL, making the number of cases small. Therefore, studies with a large number of cases are needed to verify our findings.

## Conclusions

Small intestinal MEITL is a rare cancer with atypical clinical symptoms and a poor prognosis. Diagnosis relies on histopathological examination. Characteristic immunohistochemical markers and EBER testing can confirm the diagnosis. Molecular genetics has shown that mutations in *JAK3* and *STAT5B* of the JAK/STAT pathway and inactivation of the oncogene *SETD2* play an important role in the lymphomagenesis of MEITL. Further in-depth study of the pathogenesis and discovery of additional driver genes will facilitate the use of molecularly targeted drugs as a new treatment option for MEITL.

## Data Availability

The data sets generated during and/or analyzed during the current study are available from the corresponding author on reasonable request.

## Abbreviations

MEITL: Monomorphic–epitheliotropic intestinal T-cell lymphoma
EBV: Epstein–Barr virus
TCR: T-cell receptor
NGS: Next-generation sequencing
EATL: Enteropathy-associated T-cell lymphoma
CD: Celiac disease
